# Human mitochondrial MTHFD2 is a dual redox cofactor-specific methylenetetrahydrofolate dehydrogenase/methenyltetrahydrofolate cyclohydrolase

**DOI:** 10.1186/s40170-017-0173-0

**Published:** 2017-12-06

**Authors:** Minhye Shin, Jessica Momb, Dean R. Appling

**Affiliations:** Department of Molecular Biosciences, The University of Texas at Austin, Austin, TX 78712-0165 USA

**Keywords:** MTHFD2, NADH, NADPH, One-carbon metabolism

## Abstract

**Background:**

Folate-dependent one-carbon metabolism provides one-carbon units for several biological processes. This pathway is highly compartmentalized in eukaryotes, with the mitochondrial pathway producing formate for use in cytoplasmic processes. The mitochondrial enzyme MTHFD2 has been reported to use NAD^+^ as a cofactor while the isozyme MTHFD2L utilizes NAD^+^ or NADP^+^ at physiologically relevant conditions. Because MTHFD2 is highly expressed in many cancer types, we sought to determine the cofactor preference of this enzyme.

**Results:**

Kinetic analysis shows that purified human MTHFD2 exhibits dual redox cofactor specificity, utilizing either NADP^+^ or NAD^+^ with the more physiologically relevant pentaglutamate folate substrate.

**Conclusion:**

These results show that the mitochondrial folate pathway isozymes MTHFD2 and MTHFD2L both exhibit dual redox cofactor specificity. Our kinetic analysis clearly supports a role for MTHFD2 in mitochondrial NADPH production, indicating that this enzyme is likely responsible for mitochondrial production of both NADH and NADPH in rapidly proliferating cells.

## Background

One-carbon (1C) metabolism is a universal folate-dependent pathway that generates 1C units for de novo purine and thymidylate synthesis, interconversion of several amino acids, production of universal methyl donors, and regeneration of redox cofactors. Because these metabolic processes play critical roles in cancer cells [1, 2], 1C metabolism has long been an important target for the development of chemotherapeutic drugs.

**Fig. 1:**
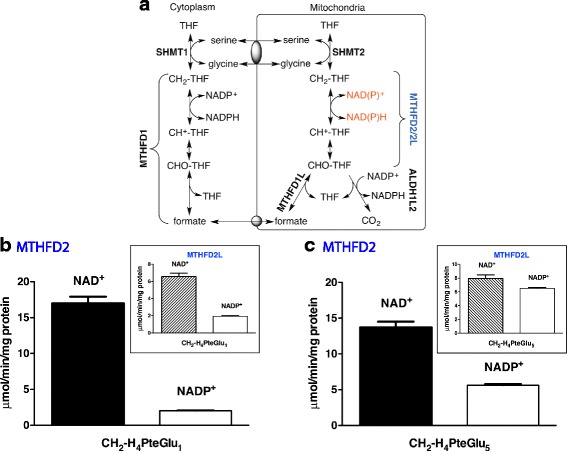
MTHFD2 exhibits NADP^+^-dependent dehydrogenase activity with 5,10-CH_2_-THF pentaglutamate. **a** Compartmentation of mammalian one-carbon metabolism. MTHFD1 is the cytoplasmic trifunctional C_1_-THF synthase that catalyzes 10-formyl-THF synthetase, 5,10-methenyl-THF cyclohydrolase, and 5,10-methylene-THF dehydrogenase activities. In mammalian mitochondria, bifunctional MTHFD2 or MTHFD2L enzymes catalyze 5,10-methenyl-THF cyclohydrolase and 5,10-methylene-THF dehydrogenase activities, and monofunctional MTHFD1L catalyzes the 10-formyl-THF synthetase reaction. SHMT1 and SHMT2 represent cytoplasmic and mitochondrial serine hydroxymethyltransferase isozymes, respectively. Gray ovals represent putative metabolite transporters. **b**, **c** Purified MTHFD2 was assayed for NAD^+^- and NADP^+^-dependent 5,10-CH_2_-THF dehydrogenase activity with saturating concentrations of CH_2_-H_4_PteGlu_1_ or CH_2_-H_4_PteGlu_5_ (insets, 5,10-CH_2_-THF dehydrogenase activity of MTHFD2L; data from ref. 11). CH_2_-H_4_PteGlu_1_ and CH_2_-H_4_PteGlu_5_ concentrations were 354 and 429 μM, respectively. NAD^+^ and NADP^+^ concentrations were 1.0 and 6.0 mM, respectively. NAD^+^-dependent reactions also included 5 mM MgCl_2_ and 25 mM P_*i*_. NADP^+^-dependent reactions included only 5 mM MgCl_2_. Each column represents the mean ± S.E. of triplicate determinations

One-carbon metabolism is highly compartmentalized in eukaryotes [3], and mitochondria play a critical role in cellular 1C metabolism. Mitochondria import 1C donors such as serine and glycine and oxidize the 1C units to formate, which is exported to the cytoplasm as a 1C unit for use in purine and thymidylate synthesis and homocysteine remethylation (Fig. 1a) [4–8]. Interconversion of 1C units in mammalian mitochondria is catalyzed by three distinct members of the MTHFD (methylene-tetrahydrofolate dehydrogenase) family of enzymes: MTHFD2L, MTHFD2, and MTHFD1L. MTHFD1L is a monofunctional 10-CHO-THF synthetase [9]. It catalyzes the final step in the mitochondrial pathway to produce formate, thus controlling the flux of 1C units from mitochondria into cytoplasmic processes.

Mitochondrial MTHFD2 and MTHFD2L are both bifunctional enzymes possessing 5,10-methenyl-THF (CH^+^-THF) cyclohydrolase and 5,10-methylene-THF (CH_2_-THF) dehydrogenase activities. MTHFD2 was initially identified as an NAD^+^-dependent 5,10-CH_2_-THF dehydrogenase [10]. The *Mthfd2* gene is expressed only in transformed mammalian cells and embryonic or nondifferentiated adult tissues [10, 11]. MTHFD2L, identified in 2011, is homologous to MTHFD2, sharing 60–65% amino acid sequence identity among various mammals. The *Mthfd2l* gene is expressed in adult mammals (highest expression in the brain and lung) and also at all stages of embryogenesis [11, 12].

*Mthfd2* has been identified in a screen of 19 cancer cell types as one of the 50 most commonly overexpressed genes [13]. Increased MTHFD2 expression is associated with acute myeloid leukemia, breast cancer, lung cancer, and liver cancer [14–20], and MTHFD2 is considered a novel target for anticancer therapy [21, 22]. A number of recent studies have shown that the mitochondrial 1C pathway is often reprogrammed in cancer cells and is especially critical for maintaining NADPH/NADP^+^ redox homeostasis [19, 23–27]. MTHFD2 is generally regarded as the enzyme responsible for this mitochondrial NADPH production, although ALDH1L2 has also been invoked [28]. Whereas ALDH1L2 clearly uses NADP^+^ [29], MTHFD2 has been considered an NAD^+^-dependent methylenetetrahydrofolate dehydrogenase since its discovery and early characterization [10, 30]. This raises the question whether MTHFD2 is in fact involved in maintaining mitochondrial NADPH/NADP^+^ redox homeostasis.

We previously showed that MTHFD2L can use either NAD^+^ or NADP^+^ at physiologically relevant substrate levels [11]. The use of NAD^+^ versus NADP^+^ will have a dramatic effect on the rate and direction of flux of 1C units in mitochondria, by affecting the equilibrium between 5,10-CH_2_-THF and 10-CHO-THF (and thus formate), depending on the relative levels of NAD^+^ and NADP^+^ in the mitochondrial matrix [11, 31]. Given the importance of MTHFD2 as a potential chemotherapeutic drug target, we have reinvestigated the redox cofactor specificity of the enzyme under more physiologically relevant conditions. We show here that MTHFD2, like MTHFD2L, possesses dual redox cofactor specificity for its CH_2_-THF dehydrogenase activity at physiologically relevant substrate levels.

## Methods

### Chemicals and reagents

NAD^+^ and NADP^+^ were purchased from US Biological (Swampscott, MA) and Sigma (St. Louis, MO), respectively. THF was prepared by the hydrogenation of folic acid (Sigma) using platinum oxide as a catalyst and purification of the THF product on a DEAE cellulose column (Sigma) [32]. CH_2_-THF was prepared nonenzymatically from THF and formaldehyde (Fisher, Waltham, MA) [33]. The yield of CH_2_-THF was determined by solving the equilibria of THF, formaldehyde, and β-mercaptoethanol [34]. Tetrahydropteroylpentaglutamate (H_4_PteGlu_5_) was prepared by a modified NaBH_4_ reduction from the corresponding pteroylpentaglutamate (PteGlu_5_) (Schircks Laboratories, Jona, Switzerland), as described previously [35]. Further preparation of 5,10-CH_2_-H_4_PteGlu_5_ was accomplished by incubation with formaldehyde as described previously [33].

### Preparation of MTHFD2 and MTHFD2L

Purified human MTHFD2 was a gift from Dr. Vipin Suri (Raze Therapeutics). Briefly, 6× histidine-tagged human MTHFD2 was expressed in *Escherichia coli* and purified using size exclusion chromatography. The resulting protein corresponded to the molecular weight of 36.7 kDa with the tag. Cloning, expression, and purification of rat MTHFD2L were conducted as described previously [11].

### 5,10-Methylene-THF dehydrogenase assay

A microplate assay was used for determination of kinetic parameters as described previously [36]. CH_2_-THF dehydrogenase activity was determined by an end-point assay. The reaction buffer consisted of 50 mM HEPES (pH 8.0), 100 mM KCl, 5 mM MgCl_2_, 0.4 mM CH_2_-THF, 40 mM β-mercaptoethanol, and either NAD^+^ (1 mM) or NADP^+^ (6 mM). Potassium phosphate (25 mM) was also included for the NAD^+^-dependent activity. Sixty microliters of reaction mixture without CH_2_-THF and 20 μl of purified MTHFD2 or MTHFD2L were mixed, and the enzyme reaction was initiated by the addition of 20 μl of CH_2_-THF followed by incubation at 30 °C for 5 min. The reaction was quenched with 200 μl of 3% perchloric acid, and the plate was read at 350 nm on FlexStation 3 (Molecular Devices, Sunyvale, CA). The path length was corrected using near-infrared measurements [37]. For the determination of kinetic parameters, initial rate data was fitted to the Michaelis-Menten equation by non-linear regression using Prism (GraphPad, La Jolla, CA).

## Results and discussion

Although the bifunctional MTHFD2 is widely known as a NAD^+^-dependent 5,10-CH_2_-THF dehydrogenase, it has been reported to use NADP^+^ at low efficiency [31]. However, that study did not use physiologically relevant substrate concentrations. To verify redox cofactor specificity of MTHFD2, we first compared 5,10-CH_2_-THF dehydrogenase activities of the enzyme with NAD^+^ and NADP^+^ under standard saturating substrate conditions. In order to allow direct comparisons between MTHFD2 and MTHFD2L, MTHFD2L activity assays were conducted in parallel. The MTHFD2L data were virtually identical to our previously published results [11]; data from [11] are included in the figures for comparison. With 5,10-CH_2_-H_4_PteGlu_1_ substrate, NAD^+^-dependent dehydrogenase activity of MTHFD2 was 8.5-fold higher than its NADP^+^-dependent activity (Fig. 1b). NAD^+^-dependent dehydrogenase activity of MTHFD2L was 3.4-fold higher than its NADP^+^-dependent activity under saturating substrate conditions (Fig. 1b; inset).

To explore redox cofactor specificity in MTHFD2 under more physiologically relevant substrate conditions, we repeated the assay with CH_2_-H_4_PteGlu_5_. Folylpolyglutamate specificity is one of the characteristic features of enzymes in the one-carbon metabolism [38], and the folate coenzymes typically found in mammalian mitochondria contain chain lengths of 6–9 glutamates [39]. While the NAD^+^-dependent activity of MTHFD2 slightly decreased, its maximal NADP^+^-dependent activity considerably increased with CH_2_-H_4_PteGlu_5_ compared to CH_2_-H_4_PteGlu_1_ (Fig. 1c). By comparison, MTHFD2L exhibits an even more dramatic increase in NADP^+^-dependent activity with the pentaglutamate coenzyme (Fig. 1c; inset).

**Fig. 2:**
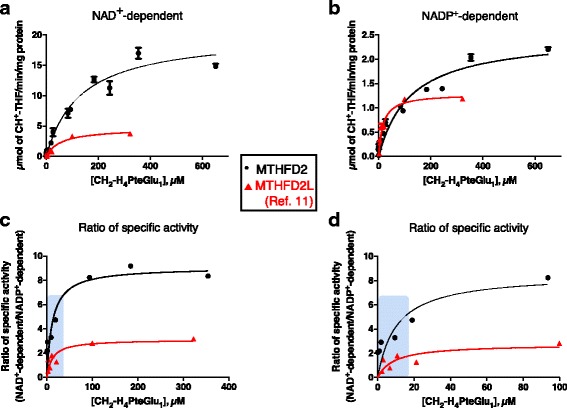
Redox cofactor specificity of MTHFD2 with CH_2_-H_4_PteGlu_1_. CH_2_-THF dehydrogenase activity of purified MTHFD2 was assayed with respect to CH_2_-H_4_PteGlu_1_ concentration using NAD^+^ (1.0 mM) (panel **a**) or NADP^+^ (6.0 mM) (panel **b**). NAD^+^-dependent reactions also included 25 mM P_*i*_. The data were fit to the Michaelis-Menten equation. **c** The ratio of NAD^+^- to NADP^+^-dependent activity plotted as a function of CH_2_-H_4_PteGlu_1_ concentration. The 0–100 μM CH_2_-H_4_PteGlu_1_ range is magnified in panel (**d**). Data for MTHFD2L from ref. [11]. The shaded boxes in **c** and **d** indicate the reported mitochondrial matrix concentration ranges for 5,10-CH_2_-THF (2.5–25 μM) [41–43]

To further investigate redox cofactor specificity of MTHFD2, steady-state kinetic parameters for CH_2_-THF dehydrogenase activity were determined using CH_2_-H_4_PteGlu_1_. MTHFD2 showed higher specific activity than MTHFD2L with both NAD^+^ and NADP^+^ (Fig. 2a, b). With the monoglutamate folate substrate, MTHFD2 exhibited a *k*_cat_/K_M_ ratio eightfold higher for NAD^+^ than for NADP^+^, indicating a strong preference for NAD^+^ at saturating substrate concentrations (Table 1). In comparison, MTHFD2L has only a twofold higher *k*_cat_/K_M_ for NAD^+^ versus NADP^+^ [11].

**Table 1:** Kinetic parameters for MTHFD2 5,10-CH_2_-THF dehydrogenase activity. 5,10-CH_2_-THF kinetic parameters were determined using saturating concentrations of NAD^+^ (1.0 mM) or NADP^+^ (6.0 mM). When NAD^+^ was used, potassium phosphate (25 mM) and MgCl_2_ (5 mM) were also included

	CH_2_-H_4_PteGlu_1_			CH_2_-H_4_PteGlu_5_		
	K_M_ (μM)	k_cat_ (s^−1^)	k_cat_/K_M_ (s^−1^ μM^−1^)	K_M_ (μM)	k_cat_ (s^−1^)	k_cat_/K_M_ (s^−1^ μM^−1^)
NAD^+^-dependent	133 ± 20	12.4 ± 0.71	0.093	359 ± 32	15.4 ± 0.55	0.043
NADP^+^-dependent	123 ± 24	1.5 ± 0.11	0.012	302 ± 35	6.4 ± 0.29	0.021

To better understand the cofactor preference of the MTHFD2 dehydrogenase activity, the ratio of NAD^+^-dependent specific activity versus NADP^+^-dependent specific activity was calculated at each CH_2_-H_4_PteGlu_1_ concentration (Fig. 2c). At high CH_2_-H_4_PteGlu_1_ concentrations, both MTHFD2 and MTHFD2L clearly preferred NAD^+^. However, as the folate substrate concentration was lowered into the physiological range (2.5–25 μM reported mitochondrial matrix CH_2_-THF concentration range), the ratio of NAD^+^- to NADP^+^-dependent activity for both enzymes decreased. MTHFD2L approached a ratio of 1, whereas the ratio for MTHFD2 dropped from 8 to 2–4 in the physiological folate range (Fig. 2d).

**Fig. 3:**
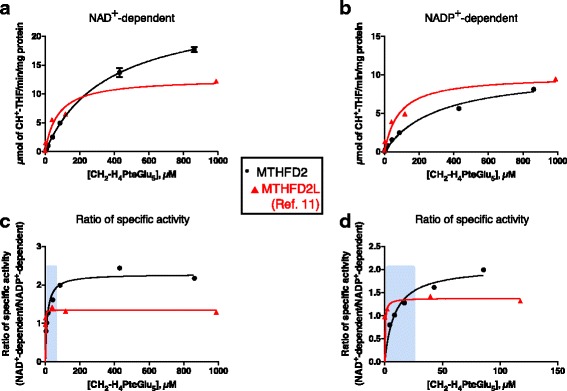
Redox cofactor specificity of MTHFD2 with CH_2_-H_4_PteGlu_5_. CH_2_-THF dehydrogenase activity of purified MTHFD2 was assayed with respect to CH_2_-H_4_PteGlu_5_ concentration using NAD^+^ (1.0 mM) (panel **a**) or NADP^+^ (6.0 mM) (panel **b**). NAD^+^-dependent reactions also included 25 mM P_*i*_. The data were fit to the Michaelis-Menten equation. **c** The ratio of NAD^+^- to NADP^+^-dependent activity plotted as a function of CH_2_-H_4_PteGlu_5_ 2concentration. The 0–150 μM CH_2_-H_4_PteGlu_5_ range is magnified in panel (**d**). Data for MTHFD2L from ref. [11]. The shaded boxes in **c** and **d** indicate the reported mitochondrial matrix concentration ranges for 5,10-CH_2_-THF as described in Fig. 2

With the more physiologically relevant pentaglutamate substrate (CH_2_-H_4_PteGlu_5_), MTHFD2’s preference for NAD^+^ is dramatically decreased (Fig. 3a, b). MTHFD2 exhibited a *k*_cat_/K_M_ ratio only twofold higher for NAD^+^ than for NADP^+^ (Table 1). Moreover, importantly, as the pentaglutamate substrate concentration was lowered into the physiological range, the ratio of NAD^+^- to NADP^+^-dependent activity for MTHFD2 approached 1 (Fig. 3c, d), similar to MTHFD2L.

## Conclusion

The role of MTHFD2 in 1C metabolism and generation of redox coenzymes places this enzyme in two pathways of central metabolic importance. Understanding how MTHFD2 is involved in both of these biological processes is of critical importance to effectively develop therapeutics targeting this enzyme for cancer treatment. As previously reported [31, 40], the methylenetetrahydrofolate dehydrogenase activity of MTHFD2 exhibits a higher preference for NAD^+^ than for NADP^+^ with monoglutamylated THF (Figs. 1b and 2). However, using pentaglutamylated THF, a physiologically relevant substrate that the enzyme would encounter in mitochondria, MTHFD2 shows increased NADP^+^-dependent activity (Figs. 1b and 3). Indeed, at the lowest CH_2_-H_4_PteGlu_5_ concentrations, MTHFD2 is more active with NADP^+^ than with NAD^+^ (Fig. 3d). These data reveal that MTHFD2, like MTHFD2L, is a dual redox cofactor-specific methylenetetrahydrofolate dehydrogenase, active with both NAD^+^ and NADP^+^ under physiological conditions.

The mitochondrial 1C pathway is now understood to be especially critical for maintaining NADPH/NADP^+^ redox homeostasis [19, 23–27]. Despite the fact that MTHFD2 has been consistently described as an NAD^+^-dependent 5,10-CH_2_-THF dehydrogenase since its initial description in 1960 [30], several of these studies invoked MTHFD2 as the source of mitochondrial NADPH production. The kinetic analyses reported here clearly reveal the ability of MTHFD2 to use NADP^+^ in vitro and provide a mechanistic basis for these flux analyses that implicate MTHFD2 in mitochondrial NADPH production [24, 26].

Both *Mthfd2* and *Mthfd2l* are expressed during embryogenesis but differ in timing of expression. *Mthfd2l* expression is low in early developmental stages but begins to increase at embryonic day 10.5 and remains elevated through birth while *Mthfd2* is expressed more abundantly during early developmental stages and begins to taper off, with little or no expression observed in most adult tissues [4, 11]. Due to the similarity of the enzymatic activities of MTHFD2 and MTHFD2L, we propose that MTHFD2 may be expressed to boost flux through the mitochondrial folate pathway during early periods of embryogenesis when MTHFD2L alone is not sufficient to support high rates of cell proliferation. Likewise, enhanced expression of MTHFD2 in cancer cells is predicted to enable increased flux through the mitochondrial 1C metabolic pathway, enabling unregulated proliferation.

## Abbreviations

10-CHO-THF: 10-Formyl-tetrahydrofolate
1C: One-carbon
CH^+^-THF: 5,10-Methenyl-tetrahydrofolate
CH_2_-H_4_PteGlu_1_: 5,10-Methylene-tetrahydropteroyl monoglutamate
CH_2_-H_4_PteGlu_5_: 5,10-Methylene-tetrahydropteroyl pentaglutamate
CH_2_-THF: 5,10-Methylene-tetrahydrofolate
HEPES: 4-(2-Hydroxyethyl)-1-piperazineethanesulfonic acid
THF: Tetrahydrofolate
